# TRIM52 plays an oncogenic role in ovarian cancer associated with NF-kB pathway

**DOI:** 10.1038/s41419-018-0881-6

**Published:** 2018-09-05

**Authors:** Weihong Yang, Li Liu, Caixia Li, Ning Luo, Rong Chen, Li Li, Fudong Yu, Zhongping Cheng

**Affiliations:** 1 Department of Gynecology and Obstetrics, Tenth People’s Hospital, Tongji University School of Medicine, Shanghai, China; 2 Gynecologic Minimally Invasive Surgery Research Center, Tongji University School of Medicine, Shanghai, China; 3 Shanghai Science Institute of Family Planning, Shanghai, China

## Abstract

Emerging evidence suggests that the members of the tripartite motif (TRIM) family play a crucial role in cancer development and progression. The purpose of the study was to explore TRIM52's role in tumorigenesis and its potential molecular mechanism in ovarian cancer. The study demonstrated that knockdown of TRIM52 in SKOV3 and CAOV3 cells inhibited ovarian cancer cell invasion, migration, and proliferation, and induced cell apoptosis. On the contrary, overexpression of TRIM52 in HO8910 cells showed contrary results. Further, overexpression of TRIM52 enhanced the expression of phosphorylated IKKβ and IKBα proteins and nuclear protein P65, which implied the activation of NF-kB signal pathway. Knockdown of TRIM52 downregulated the mRNA and protein levels of NF-kB signal downstream effectors of the NF-kB pathway, including MMP9, Bcl2, IL8, and TNFα, but upregulated caspase-3 expression. These results suggested that activation of the NF-kB pathway is involved in TRIM52-mediated regulation in ovarian cancer. The nude mice study further confirmed that knockdown of TRIM52 blocked tumor growth, inhibited cell proliferation, and promoted cell apoptosis. Our data strongly suggested that TRIM52 plays an oncogenic role in ovarian cancer development associated with the NF-kB signal pathway and may be a potential target for cancer therapy.

## Introduction

Ovarian cancer is the most lethal tumor in gynecologic malignancy and causes about 125,000 deaths globally per year^1^. Although there have been advances in surgery and chemotherapy protocols, overall prognosis remains relatively poor. Late detection, intrinsic and acquired chemoresistance, and remarkable heterogeneity are mainly responsible for these clinical outcomes^2^. Due to the progressive study of molecular genetics, cancer has been regarded as a genetic disease^3^. The precise treatment targeting genes associated with the regulation in tumor growth and progression is getting more and more attention^4–7^. It is necessary to carry out researches to identify the novel diagnosis marker or treatment target involved in tumorigenic regulation in ovarian cancer.

The tripartite motif (TRIM) family is composed of genes that encode proteins containing TRIM. The integrated module comprised three different types of domains: RING domain (R), B-box domain (B), and a coiled-coil (CC) region (RBCC). The TRIM protein family is found to be involved in a wide range of biological processes, such as cell growth, development, and cellular differentiation^8,9^. Emerging evidence suggests that TRIM proteins play a crucial role in cancer development^10^. TRIM25/EFP (estrogen-responsive finger protein) was found to be highly expressed in breast cancer^11^. EFP functions as an E3-Ub ligase and directly degrades the cell cycle regulatory protein 14-3-3σ, which leads to cell cycle progression and tumor growth. Under stress conditions, upregulated TRIM8 inhibits cell proliferation by promoting the capacity of p53 to activate genes involved in cell cycle arrest and DNA repair^12^. TRIMI9/PML facilitates p53-Thrl 8 phosphorylation in response to DNA damage^13^. TRIM24 deletion in human breast cancers leads to p53-dependent apoptosis^14^. TRIM proteins may provide novel targets for effective cancer therapies in the future.

TRIM52 as a member of TRIM family was seldom reported about its biological function. In the study, we analyzed the expression of TRIM52 in ovarian cancer and its effects on ovarian tumor growth and progression. The purpose of this study was to explore TRIM52's role in the tumorigenesis and its potentially involved molecular mechanism in ovarian cancer.

## Results

### TRIM52 expression in ovarian cancer

**Fig. 1:**
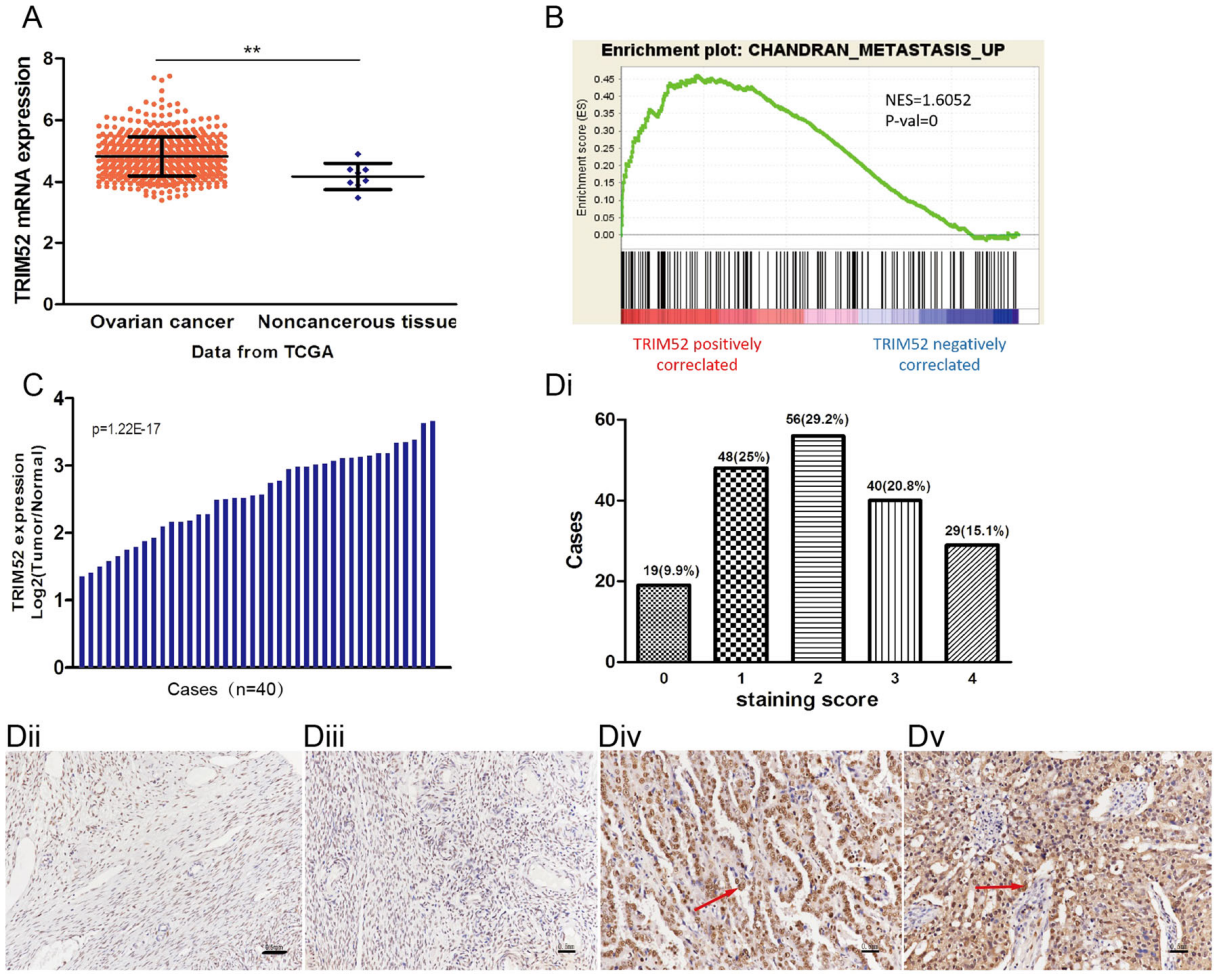
TRIM52 expression in ovarian cancer. **a** TRIKM52 expression in 568 ovarian cancer samples based on high-throughput RNA-sequencing data from TCGA, compared with noncancerous tissue (**P < 0.01). **b** The data from GESA showed that TRIM52 expression positively correlated with gene signatures involved in ovarian cancer metastasis. **c** The mRNA level of TRIN52 in 40 pairs of ovarian cancer and normal tissue was detected by qPCR. **d** Tissue microarray (TMA) study was employed to assess TRIM52 expression. The staining scores decided by immunohistochemistry experiment listed in **di**. **dii**, **diii**, **div** and **dv** showed the positive staining cells with the percentages of 5–25, 25–50, 50–75, and >75%, respectively

We analyzed the expression of TRIM52 in ovarian cancer based on high-throughput RNA-sequencing data from The Cancer Genome Atlas project (TCGA, https://tcga-data.nci.nih.gov/tcga/), including 568 ovarian cancers samples and eight normal tissue samples. As shown in Fig. 1a, TRIM52 expression in tumor tissue was significantly higher compared with normal tissue (*P* < 0.01). GESA confirmed that TRIM52 expression positively correlated with gene signatures involved in ovarian cancer metastasis pathway (Fig. 1b). The results implied that TRIM52 was closely associated with ovarian cancer development.

The specimens from 40 ovarian cancer cases were collected. TRIM52 mRNA levels detected by qPCR were higher in ovarian cancer tissue than in paired noncancerous tissue. The values of log2 (tumor/normal) from all paired specimens have shown to be positive (Fig. 1c). Tissue microarray (TMA), containing 192 ovarian cancer cases and eight normal ovary cases, was employed to evaluate TRIM52 expression. IHC assay revealed that TRIM52 was stained positively in cell nucleus and cytoplasm. The positive staining percentages of 5–25, 25–50, 50–75, and >75% were defined as scores 1, 2, 3, and 4 (Fig. 1dii, iii, iv, v), respectively. In total, 90.1% of the sample with the score of 1–4 presented positive staining (Fig. 1di).

### Construct stable cell lines with RNA interference

PCR experiments showed that relative TRIM52 mRNA level in SKOV3 and CKOV3 cells was higher than that in the other three cells. The lowest level of TRIM52 mRNA was found in HO8910 cells (Supplement 1A). Analysis of TRIM52 protein expressions in diverse cell lines supported similar results that higher TRIM52 protein levels were detected in SKOV3 and CAOV3 cells and lowest protein expressions were detected in HO8910 cells (Supplement 1B). Immunofluorescence assay further confirmed that green fluorescence staining implying TRIM52 expression was frequently found in SKOV3 and CKOV3 cells. However, there were few staining cells in HO8910 cells (Supplement 1C). The observation from immunofluorescence assay was consistent with the results of qPCR and western blot (WB) experiments.

SKOV3 and CAOV3 cells were determined to be infected with shRNA–TRIM52 lentivirus. Nonspecific scramble shRNA sequence was served as the negative control (NC). HO8910 was infected with synthetic TRIM52 lentivirus and empty lentiviral vector served as control. TRIM52 mRNA and protein expressions were remarkably decreased in SKOV3 and CAOV3 cells treated by TRIM52-Ri1,2, and 3 compared with NC cells (Supplement 1D–G). Conversely, TRIM52 expressions enhanced in HO8910 cells were incubated with synthetic TRIM52 lentivirus (Supplement 1H, I). Considering the efficiency of RNA interference, TRIM52-Ri2 and 3 were chosen for following assays. The synthetic TRIM52 lentivirus was employed to infect HO8910 cells for next assays involvingTRIM52 overexpression.

### Silence of TRIM52 inhibits ovarian cancer cell invasion, migration, and proliferation, but induces cell apoptosis

**Fig. 2:**
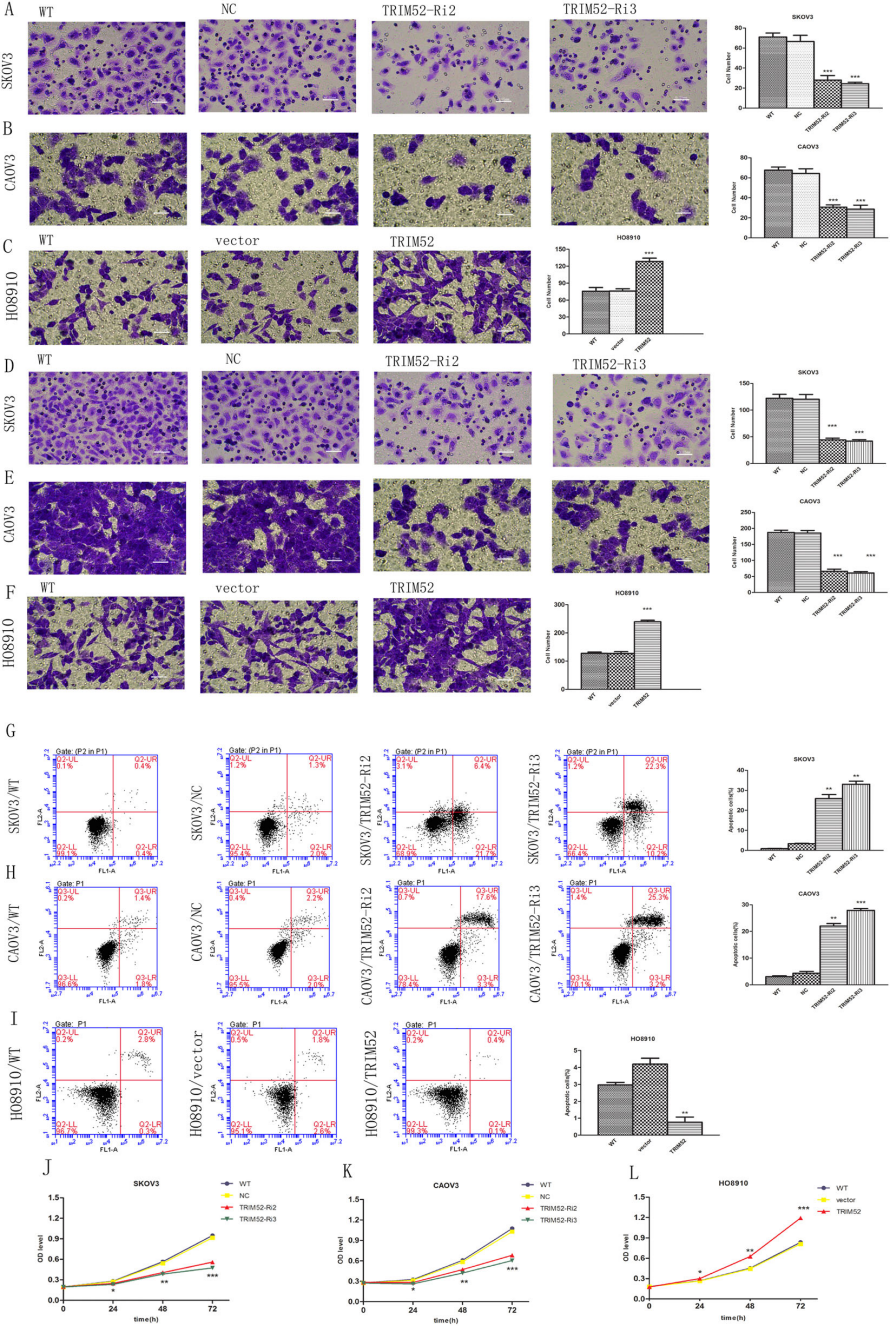
TRIM52-mediated regulation in ovarian cancer cells invasion, migration, proliferation, and apoptosis. Transwell experiments were employed to evaluate the effects of knockdown of TRIM52 in SKOV3/CAOV3 cells and overexpression of TRIM52 in HO8910 cells in ovarian cancer cell invasion (**a**–**c**) and migration (**d**–**f**). Annexin V-fluorescein isothiocyanate (FITC) and PI double-staining and Flow cytometry were performed to detect cell apoptosis in treated SKOV3/CAOV3 cells (**g**, **h**) and infected HO8910 cells (**i**). Cell Count Kit-8 (CCK-8) was used to assess cell proliferation. Optical density values (OD) at 24 h, 48 h, and 72 h after transfection were recorded in SKOV3/CAOV3 cells (**j**, **k**) and HO8910 cells (**l**). *P < 0.05, **P < 0.01, ***P < 0.001, versus NC or vector

We evaluated cell invasion and migration by Transwell assays. Four experimental groups were built containing untreated cells (wild cells, WT), infected cell lines with TRIM52-Ri2,3 and scramble NC. The total invading cells declined by 58.21 and 64.18% in infected SKOV3 cells with TRIM52-Ri2 and TRIM52-Ri3, respectively (Fig. 2a). In treated CAOV3 cells by TRIM52-Ri2,3, invading cells reduced by 51.56 and 54.69% (Fig. 2b). Similarly, Transwell assays showed that the amount of migrating cells obviously decreased in treated SKOV3 and CAOV3 cells by TRIM52-Ri2,3 (Fig. 2d, e). It suggested that knockdown of TRIM52 downregulated the ability of ovarian cancer cell invasion and migration.

Cell apoptosis was detected by flow cytometry assay. The number of apoptotic cells notably increased in TRIM52-knockdown SKOV3 and CKOV3 cells compared with NC (Fig. 2g, h).

To evaluate cell proliferation, CCK-8 experiment was carried out and OD values from different treated cells were obtained. Optical density (OD) values remarkably decreased in TRIM52-knockdown SKOV3 and CKOV3 cells at 24 h, 48 h, and 72 h after infecting, relative to NC (Fig. 2j, k). These results indicated that TRIM52 knockdown induced ovarian cancer cell apoptosis and inhibited cell proliferation.

**Fig. 3:**
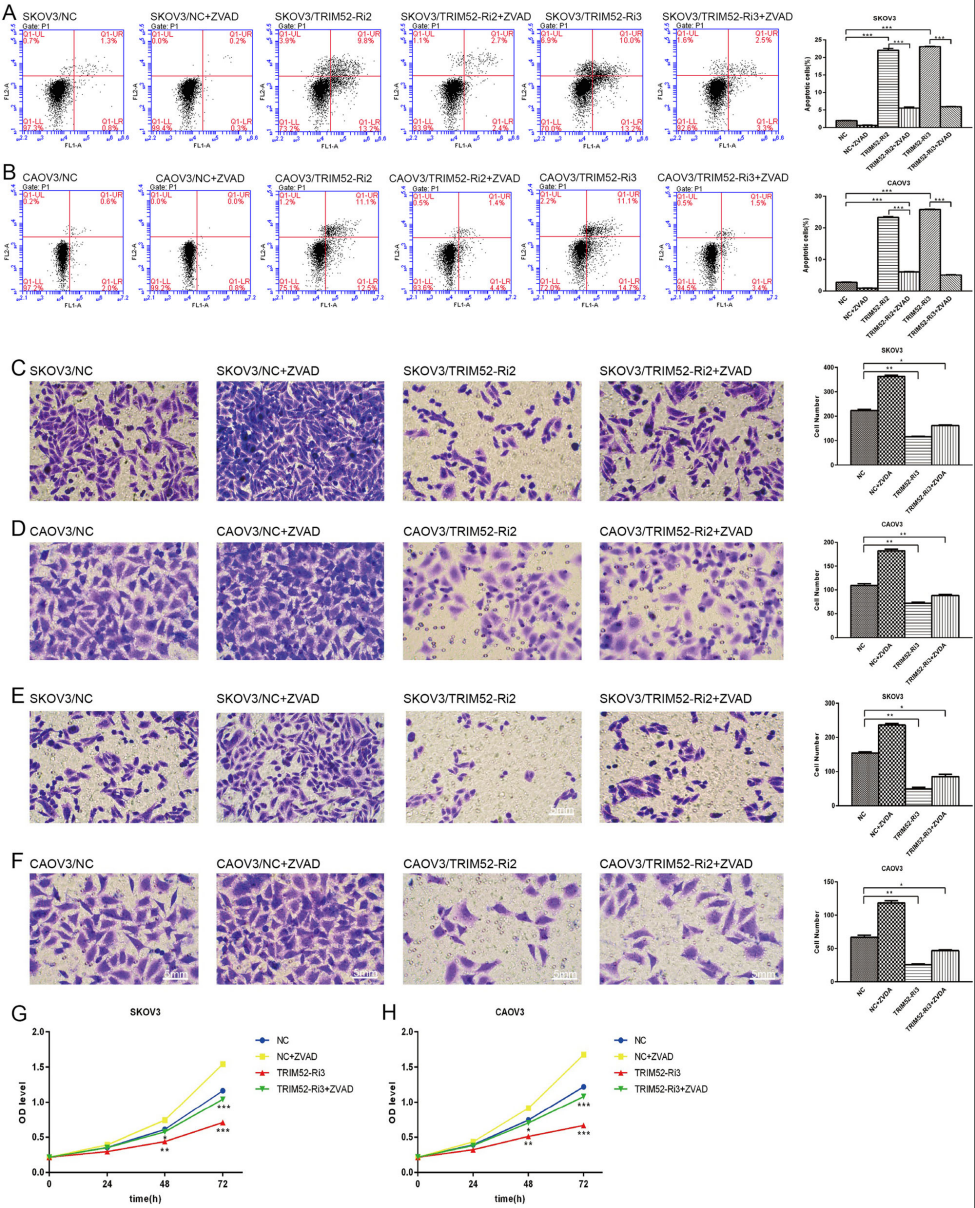
ZVAD control experiments. After adding ZVAD in SKOV3 and CAOV3 cells treated by TRIM52-Ri2 or TRIM52-Ri3, flow cytometry were performed to detect cell apoptosis (**a**, **b**). Transwell experiments were employed to evaluate cell migration and invasion in infected SKOV3 cells plus ZVAD (**c**, **e**) and infected CAOV3 cells plus ZVAD (**d**, **f**). In treated SKOV3 and CAOV3 cells with the addition of ZVAD, OD values obtained by CCK-8 assays at 24 h, 48 h, and 72 h after transfection were recorded (**g**, **h**). *P < 0.05, **P < 0.01, ***P < 0.001, versus NC

ZVAD (an apoptosis inhibitor) was added as a control in the experiment. Additional ZVAD blocked the increasing of cells apoptosis mediated by knockdown TRIM52 (Fig. 3a, b). Cells invasion and migration, as well as cells proliferation still significantly decreased in treated SKOV3/CAOV3 by downregulated TRIM52, even though ZVAD was added (Fig. 3c–h). It further indicated that knockdown of TRIM52 inhibited ovarian cancer cell invasion, migration, and proliferation, but promoted cell apoptosis.

### TRIM52 overexpression promotes ovarian cancer cell invasion, migration, and proliferation, but inhibits cell apoptosis

HO8910 cells were treated by synthetic TRIM52 lentivirus or empty lentiviral vector or DEME medium. Transwell assays showed that the count of invading or migrating cells obviously increased in infected HO8910 than in control vector (Fig. 2c, f). Flow cytometry assay revealed that the number of apoptotic cells remarkably decreased in overexpressing-TRIM52 HO8910 cells (Fig. 3i). But OD values of CCK-8 experiment implying cell proliferation increased at 24 h, 48 h, and 72 h after HO8910 cells were infected by TRIM52 lentivirus compared with the vector (Fig. 2l). The results suggested that TRIM52 overexpression promoted ovarian cancer cell invasion, migration, and proliferation, but inhibited cell apoptosis.

### TRIM52 expression promotes the activation of NF-Kb signal pathway in ovarian cancer

**Fig. 4:**
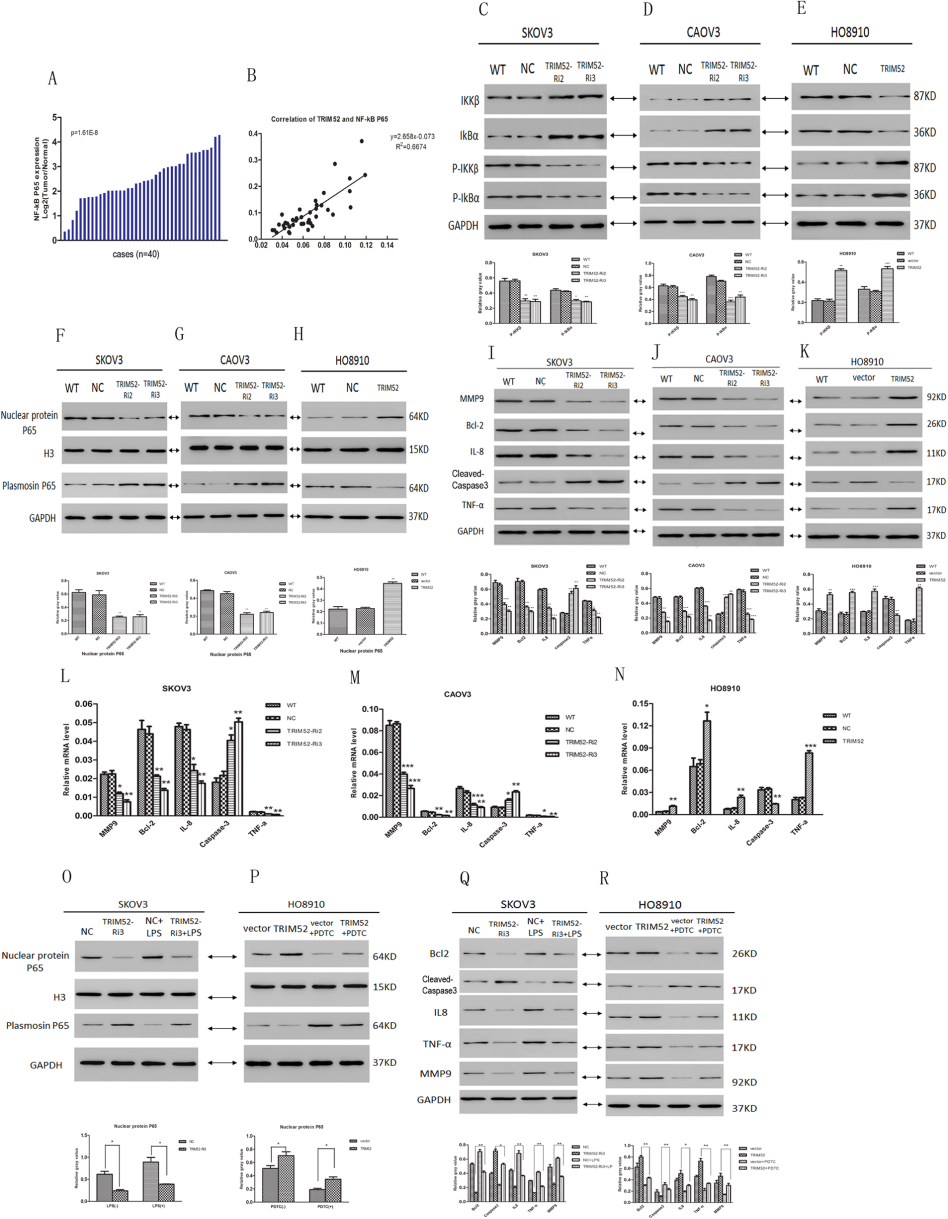
TRIM52 expression promoted the activation of NF-kB signal pathway in ovarian cancer. **a** The mRNA level of NF-kB P65 in 40 pairs of ovarian cancer and normal tissue was detected by qPCR. Positive log_2_ (tumor/normal) on the y-axis indicated increasing expression of NF-kB P65 in a tumor tissue. **b** Pearson’s correlation analysis of TRIM52 and NF-kB P65 in 40 ovarian cancer specimens. Proteins levels of IKKβ and IKBα and phosphorylated IKKβ and IKBα were evaluated by western blot in treated SKOV3/CAOV3 cells with TRIM52-Ri2,3 (**c**, **d**) and in infected HO8910 cells by synthetic TRIM52 lentivirus (**e**). Nuclear protein P65 expressions were detected by WB similarly in treated SKOV3/ CAOV3 cells (**f**, **g**) and in infected HO8910 cells (**h**). Protein levels of NF-kB downstream effectors including MMP9, Bcl2, caspase 3, IL8, and TNFα were detected by WB in SKOV3/CAOV3 cells with TRIM52 knockdown (**i**, **j**) and in HO8910 cells infected with TRIM52 lentivirus (**k**). mRNA expressions of MMP9, Bcl2, caspase 3, IL8, and TNFα were confirmed by qPCR in treated SKOV3/CAOV3 cells with TRIM52-Ri2,3 (**l**, **m**) and in HO8910 cells infected with TRIM52 lentivirus (**n**). Nuclear protein P65 and plasmosin P65 protein expressions were evaluated by WB in SKOV3 cells treated by TRIM52-Ri3 plus LPS (an activator of NF-kB P65) (**o**) and in HO8910 cells treated by TRIM52 lentivirus plus PDTC (an inhibitor of NF-kB P65) (**p**). Proteins expressions of MMP9, Bcl2, caspase 3, IL8, and TNFα were confirmed by WB in TRIM52-knockdown SKOV3 cells with LPS (**q**) and overexpressing-TRIM52 HO8910 cells with PDTC (**r**). *P < 0.05, **P < 0.01, ***P < 0.001, versus NC or vector

Emerging evidence suggests that TRIM family proteins are involved in the regulation of the NF-κB pathway and associated with various pathologies like cancer, inflammatory, and autoimmune disorders^15^. To explore whether TRIM52 expression contributed to the activation of NF-KB pathway in ovarian cancer, NF-kB P65 mRNA level as well as the correlation between TRIM52 and NF-kB P65 were analyzed. As showed in Fig. 4a, NF-kB P65 mRNA expressions increased in all 40 EOC. Pearson’s correlation analysis revealed that NF-kB P65 was significantly positively correlated with TRIM52 (r^2^ = 0.6674) (Fig. 4b).

Then, the protein levels of IKBα (NF-kB inhibitor α), IKKβ (IKB kinase β), and phosphorylated IKKβ and IKBα protein expressions (p-IKKβ, p-IKBα) were detected by western blot. The results showed that p-IKKβ and p-IKBα proteins were downregulated in SKOV3 and CAOV3 infected by TRIM52-Ri2,3, but upregulated in HO8910 cells infected by TRIM52 lentivirus (Fig. 4c-e).

To further identify the activation of NF-kB signal pathway, nuclear protein P65 and plasmosin P65 were tested. Nuclear protein P65 expression obviously decreased in knockdown TRIM52 SKOV3 and CKOV3 cells (Fig. 4f, g). Conversely, increasing nuclear protein P65 level was found in overexpressing-TRIM52 HO8910 cells (Fig. 4h). Moreover, we assessed mRNA and protein expressions of NF-kB downstream effectors containing MMP9, Bcl2, caspase 3, IL8, and TNFα. Knockdown of TRIM52 in SKOV3 and CAOV3 cells downregulated the mRNA and protein levels of MMP9, Bcl2, IL8, and TNFα and upregulated caspase-3 expression (Fig. 4i, j, l, m). Overexpression of TRIM52 in HO8910 cells contributed to the contrary results (Fig. 4k, n). It was strongly suggested that TRIM52 induced the activation of NF-kB signal pathway in ovarian cancer.

In addition, LPS (an activator of NF-kB P65) or TNF-α were co-incubated with SKOV3 cells infected by TRIM52-Ri3 lentivirus. PDTC (an inhibitor of NF-kB P65) was added into HO8910 cells infected by synthetic TRIM52 lentivirus. Knockdown TRIM52 SKOV3 cells notably weakened the LPS-induced upregulation in nuclear protein P65 level (Fig. 4o). Similarly, nuclear protein P65 expression was compromised by the silence of TRIM52 (Supplement 2A, B). But overexpression TRIM52 in HO8910 cells reversed the PDTC-mediated downregulation in nuclear protein P65 expression (Fig. 4p). It strongly indicated that nuclear protein P65 level implying the activation of NF-kB signal was closely correlated with TRIM52 gene expression.

Furthermore, protein expressions of NF-kB downstream effectors including MMP9, Bcl2, IL8, and TNFα were upregulated, but caspase 3 was downregulated in TRIM52-knockdown SKOV3 cells with additional LPS (Fig. 4q). Conversely, MMP9, Bcl2, IL8, and TNFα proteins were downregulated, and caspase-3 protein was upregulated in TRIM52-overexpression HO8910 cells with additional PDTC compared to without additional PDTC (Fig. 4r). It indicated that MMP9, Bcl2, and caspase 3 may play roles on TRIM52-mediated regulation in ovarian cancer cells invasion, migration, and apoptosis. Inflammatory cytokine IL8 and chemokine TNFα implied the activation of NF-kB pathway.

### NF-kB signal pathway involves TRIM52-mediated regulation in ovarian cancer cells invasion, migration, apoptosis, and proliferation

**Fig. 5:**
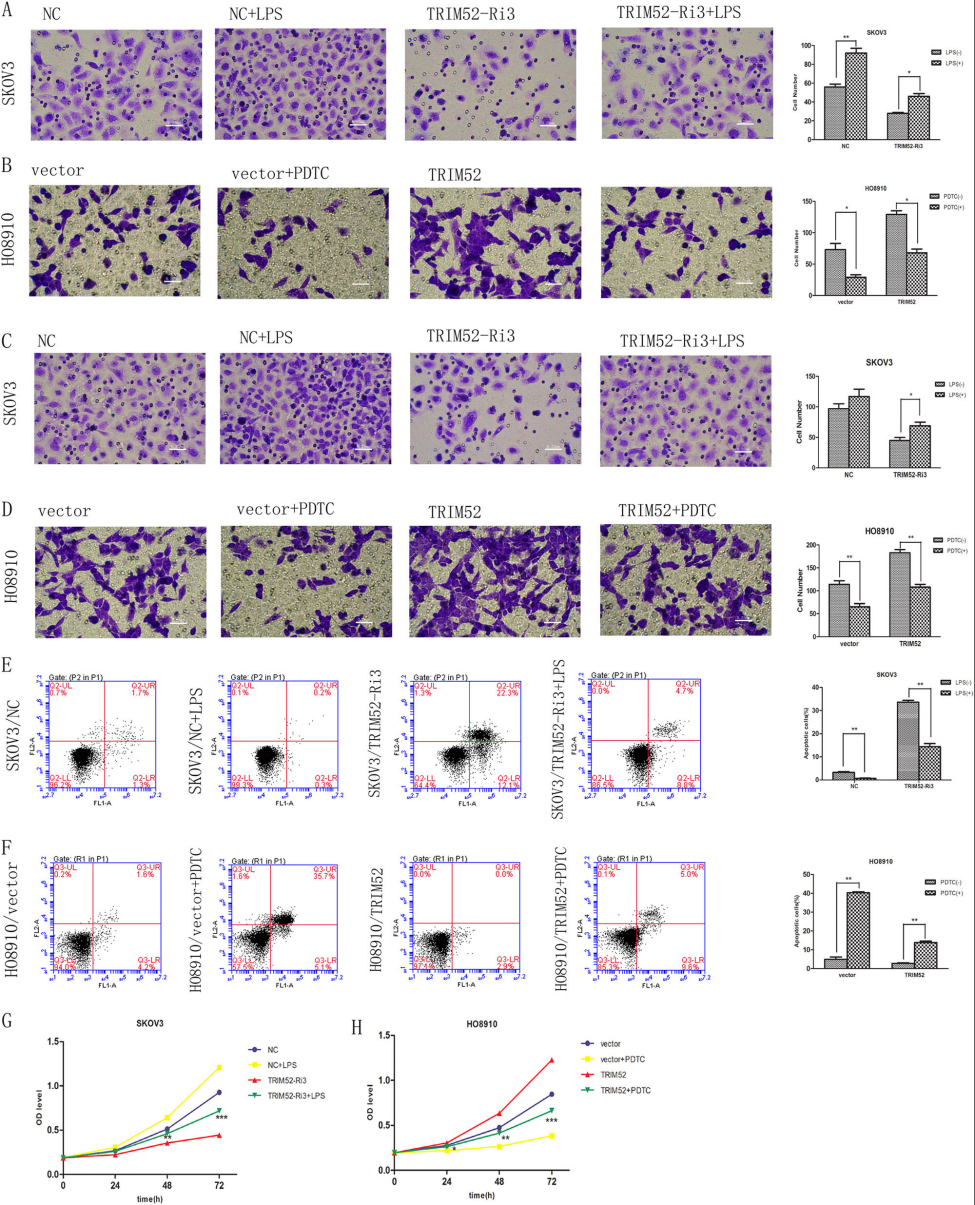
NF-kB pathway involved TRIM52-mediated regulation in ovarian cancer cell invasion, migration, apoptosis, and proliferation. Transwell assays were employed to evaluate cells invasion and migration in SKOV3 cells treated with TRIM52-Ri3 plus LPS (**a**, **c**) and in HO8910 cells treated with TRIM52 lentivirus plus PDTC (**b**, **d**). Cell apoptosis were assessed by Annexin V-fluorescein isothiocyanate (FITC) and PI double-staining and flow cytometry in SKOV3 cells treated with TRIM52-Ri3 plus LPS (**e**) and in HO8910 cells treated with TRIM52 lentivirus plus PDTC (**f**). CCK-8 experiments were used to evaluate cell proliferation in SKOV3 cells treated with TRIM52-Ri3 plus LPS (**g**) and in HO8910 cells treated with TRIM52 lentivirus plus PDTC (**h**). *P < 0.05, **P < 0.01, ***P < 0.001, (**g**: TRIM52-Ri3 versus TRIM52-Ri3 + LPS; **h**: TRIM52 versus TRIM52 + PDTC)

Transwell assay showed that adding LPS into SKOV3 cells, treated by RNA interference, reversed knockdown-TRIM52 induced downregulation in cell invasion and migration (Fig. 5a, c). Adding PDTC into infected HO8910 cells alleviated overexpressing-TRIM52 mediated upregulation in cell invasion and migration (Fig. 5b, d). It suggested that NF-kB pathway activation was closely correlated with TRIM52-mediated regulation in cell invasion and migration.

With the addition of LPS, total apoptotic cells were alleviated in TRIM52-knockdown SKOV3 cells (Fig. 5e). But additional PDTC in infected HO8910 cells reversed overexpressing TRIM52-mediated downregulation in apoptotic cells (Fig. 5f). It indicated that NF-kB activation involved TRIM52-mediated regulation in cell apoptosis.

CCK-8 assay showed that adding LPS into TRIM52-knockdown SKOV3 cells increased the OD value at 48 h and 72 h after co-incubation (Fig. 5g). But additional PDTC decreased the OD value in TRIM52-overexpression HO8910 cells at 24 h, 48 h, and 72 h after infection (Fig. 5h). It suggested that NF-kB activation was connected with TRIM52-mediated regulation in cell proliferation.

### Knockdown TRIM52 in SKOV3 cells inhibits tumor growth and promotes tumor cells apoptosis in vivo

**Fig. 6:**
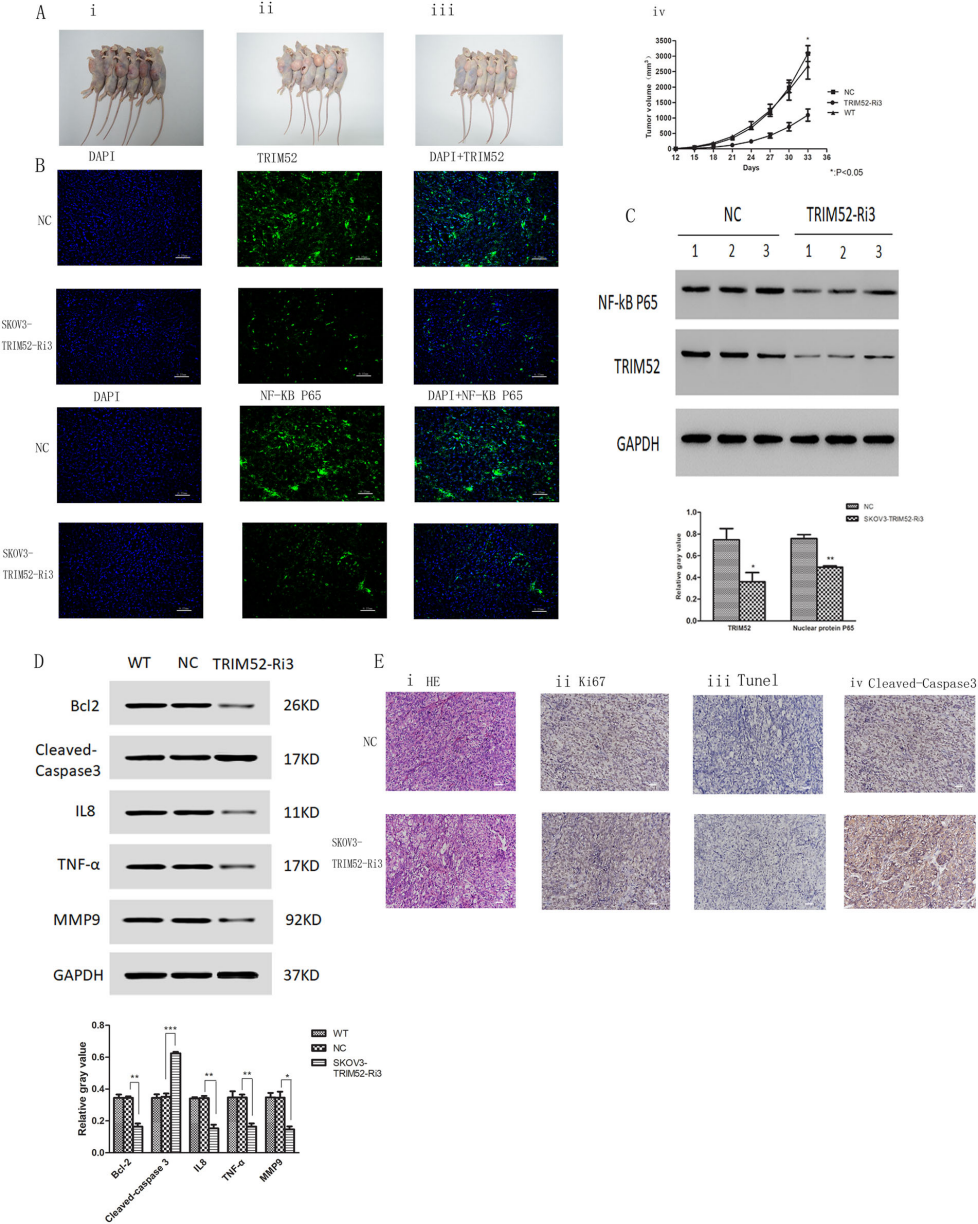
Knockdown TRIM52 inhibited tumor growth and promoted tumor cells apoptosis in vivo. Treated SKOV3 cells with TRIM52-Ri3 and no scramble sequence as negative control (NC) as well as noninfected SKOV3 cells (WT) were injected into the mouse axilla at 4 × 10^6^ each, respectively. After 33 days post inoculation, mice were killed and tumor formation was shown in **ai** (WT), **aii** (NC), and **aiii** (TRIM52-knockdown SKOV3 cells). The curves based on tumor growth were described in Aiv. Immunofluorescence assays were employed to evaluate the TRIM52 and NF-kB P65 expressions in tumor specimens (**b**). TRIM52 and NF-kB P65 proteins expressions of tumor specimens from six mice were evaluated by western blot (**c**). Protein expressions of MMP9, Bcl2, caspase 3, IL8, and TNFα were detected by western blot (**d**). HE staining revealing TRIM52 expressions of tumor specimens were shown in **ei**. Tumor cells proliferation and apoptosis were assessed by Ki-76 immumohistochemical (IH) experiment (**eii**) and Tunnel staining (**eiii**), respectively. Cleaved caspase-3 staining was performed to confirm the effect of knockdown TRIM52 on cell apoptosis (**eiv**). *P < 0.05, **P < 0.01, ***P < 0.001, versus NC

We assessed the effect of TRIM52 in tumor formation in athymic nude mice by subcutaneously injecting SKOV3 cells. The volumes of tumor were recorded from 12th to 33rd day after inoculation. As shown in Fig. 6ai-iv, the size of the tumor was much smaller in the mice group injected with SKOV3 cells treated with TRIM52-Ri3 lentivirus than those of nontargeted RNA control and noninfected cells group. It clearly suggested that knockdown of TMEM52 inhibited tumor growth in mice.

Immunofluorescence staining was employed to evaluate TRIM52 and NF-kB P65 expressions in mice tumor. The tumor slide showed less positive green fluorescence cells in the group treated by TRIM52-knockdown SKOV3 cells compared with NC (Fig. 6b), which implied that TRIM52 knockdown downregulated TRIM52 and NF-kB P65 expressions. Western blot confirmed that TRIM52 and NF-kB P65 protein levels decreased in TRIM52-knockdown tumor (Fig. 6c). The alteration of expressions of NF-kB downstream effectors determined by western blots on the tumor lysates was shown in Fig. 6d.

In addition, HE staining and Ki-76 immumohistochemical experiment were performed to evaluate the tumor growth. The results showed that positive staining cells decreased in the tumor treated by TRIM52 knockdown compared with NC (Fig. 6ei, ii). Tunnel staining assay as well as caspase-3 staining revealed that the amount of apoptotic cells was enhanced in the TRIM52-knockdown tumors (Fig. 6eiii, iv). It indicated that knockdown of TRIM52 inhibited tumor cell growth and proliferation, but promoted cell apoptosis.

## Discussion

In the study, we firstly analyzed the TRIM52 expression in ovarian cancer tissue. TRIM52 mRNA level in tumor tissue was higher than that of paired noncancerous tissue (Fig. 1c). TRIM52 Immunohistochemistry (IHC) assays based on TMA showed obviously positive staining in cell nucleus and cytoplasm of the tumor samples (Fig. 1dii-v). It indicated that TRIM52 played an oncogenic role in ovarian cancer and was linked to tumor growth and development.

It is well known that members of the TRIM protein family are found in all multicellular eukaryotes and function in a wide range of cellular processes, such as cell growth, development, and differentiation. This is not surprising that alteration of these proteins can affect transcriptional regulation, cell proliferation, and apoptosis, which is implicated in regulating carcinogenesis^10^. To explore the role of TRIM52 in tumorigenesis in ovarian cancer, we performed the present study by knocking down TRIM52 in high-expressing TRIM52 SKOV3 and CAOV3 cells and overexpressing TRIM52 in low-expression TRIM52 HO8910 cells. The results showed that knockdown of TRIM52 in SKOV3 and CAOV3 cells decreased cell invasion, migration, and proliferation, but enhanced cell apoptosis (Fig. 2). Conversely, overexpression of TRIM52 in HO8910 cells promoted cell invasion, migration, and proliferation, but inhibited cell apoptosis (Fig. 2). Additional ZVAD experiments reduced the risk of spontaneous cells apoptosis mediated by knocking down TRIM52 and further verified the effectors of TRIM52 in promoting ovarian cancer cells invasion, migration, and proliferation. The study of tumorigenesis in vivo also confirmed that blocking TRIM52 inhibited tumor growth and cell proliferation, but induced cell apoptosis (Fig. 6a).

Existing studies reported that TRIMs play a critical role in the regulation of the NF-kB pathway^15–17^. TRIM20, known as MEFV (Mediterranean fever) protein, regulates the turnover of IκBα and interaction with NF-kB p65, and positively regulates the NF-kB pathway^18^. TRIM19 acting as transcriptional repressor of NF-kB sequesters NF-kB in the nucleus to the PML-nuclear bodies in response to TNF^19^. To identify whether NF-kB pathway was involved in TRIM52-mediated regulation in ovarian cancer development, we observed the activity of the NF-kB signal pathway. In HO8910 cells treated by synthetic TRIM52 lentivirus, phosphorylated IKKβ and IκBα as well as nuclear protein P65 expressions were significantly upregulated compared with control cells (Fig. 4e, h). Conversely, these proteins levels notably reduced in SKOV3 and CAOV3 cells with the knockdown of TRIM52 (Fig. 4c, d, f, g). Furthermore, knockdown of TRIM52 in SKOV3 cells weakened the LPS-induced high expression of the nuclear protein P65 (Fig. 4o). Overexpression of TRIM52 in HO8910 cells reversed the PDTC-mediated low expression of the nuclear protein P65 (Fig. 4p). In addition, the study revealed that TNFα-inducing NF-kB activation was compromised by the silence of TRIM52 (Supplement 2). These results strongly suggested that TRIM52 was a positive regulator of the NF-kB pathway. Then, we identified the mRNA and protein levels of TNFα and IL8, which were the important proinflammatory cytokines in response to NF-kB signal activation. The results revealed that blocking TRIM52 decreased the expressions of TNFα and IL8, but overexpression of TRIM52 increased the levels of TNFα and IL8 (Fig. 4i-n). So, this further demonstrated that TRIM52 closely connected with the activation of NF-kB P65 and played an important role in the regulation of the NF-kB signal pathway.

To further explore whether NF-kB signal pathway is involved in TRIM52-mediated regulation in tumorigenesis in ovarian cancer, NF-kB pathway downstream effectors of MMP9, Bcl2, and caspase 3 were detected. Knockdown of TRIM52 downregulated MMP9 and Bcl2 mRNA and protein expressions, and upregulated caspase-3 expressions. Overexpression of TRIM52 obtained the reverse results (Fig. 4i-n). MMP9 is considered to play an important role in degrading extracellular matrix and is associated with invasive, aggressive, and metastatic^20^ tumors. Bcl2 and caspase 3 levels were closely linked to regulation in cell apoptosis^21,22^. The changes of downstream effectors expressions supported that NF-kB pathway contributed to TRIM52-induced regulation in cell invasion, migration, and apoptosis. Moreover, adding LPS reversed the decline of Bcl2 and MMP9, and weakened the increase of caspase 3 in knockdown TRIM52 SKOV3 cells (Fig. 4q). Additional PDTC weakened the increase of Bcl2 and MMP9 and decrease of caspase 3 in overexpressing TRIM52 HO8910 cells (Fig. 4r). It further suggested that NF-kB signal pathway involved the mechanism associated with TRIM52-mediated regulation in ovarian cancer development. In addition, adding LPS into knockdown-TRIM52 SKOV3 cells reversed the TRIM52-mediated downregulation in cell invasion, migration, and proliferation, and the upregulation in cell apoptosis (Fig. 5a, c, e, g). On the contrary, additional PDTC in overexpressing-TRIM52 HO8910 cells alleviated the TRIM52-mediated upregulation in cell invasion, migration, and proliferation, and the downregulation in cell apoptosis (Fig. 5b, d, f, h). It also strongly indicated that the activation of NF-kB impacted TRIM52-mediated regulation in tumorigenesis in ovarian cancer.

Documents have been reported that some members of the TRIM protein family are thought to be important regulators of carcinogenesis^10,17,23–27^. The famous promyelocytic leukemia (PML) gene, which encodes the protein TRIM19, is involved in the t(15;17) translocation that specifically occurs in acute promyelocytic leukemia (APL)^19^. Other members of the TRIM family are linked to cancer development possibly through ubiquitination or loss of tumor suppression functions^15^. Our study supported that TRIM52 acted as a positive regulator of tumorigenesis associated with ovarian cancer development via the activiation of the NF-kB signal pathway. But the definite molecular mechanism underlying the involvement of the NF-kB pathway is still under investigation. TRIM8-mediated positive regulation of NF-kB is mediated through the K-63-linked polyubiquitination of TAK1 in the presence of TNFα and IL1β^28^. TRIM23 interacts with HCMV (human cytomegalovirus) genome-encoded UL144 and regulates its interaction with TRAF6 to positively regulate the NF-kB signaling^29^. The system regulation of NF-kB pathway mediated by TRIM52 is not well understood. Further understanding of TRIM521 protein role in tumorigenesis and regulation of NF-kB signal pathway may provide new insights into the effective therapies in ovarian cancer.

In summary, our study confirmed that TRIM52 mRNA and protein expressions increased in ovarian cancer compared with normal ovarian tissue. This study provides the first evidence that TRIM52 may play an oncogenic role in ovarian cancer to promote cell invasion, migration, proliferation, but inhibit cell apoptosis. Moreover, our results strongly supported that the NF-kB pathway was activated by the overexpression of TRIM52 via classical activating pathway and involved in the TRIM52-mediated regulation of tumorigenesis in ovarian cancer. These findings suggested that TRIM52 may be a novel target for ovarian cancer treatment in the future.

## Methods

### Bioinformatics

TRIM52 gene expression data were obtained from The Cancer Genome Atlas (TCGA, http://tcgadata.nci.nih.gov/tcga/). The analysis contained 568 ovarian cancers and eight normal ovarian tissues. TMEM52 expression was verified to be statistically normally distributed by Shapiro–Wilk test. Statistical significance is based upon unpaired *t* test.

To explore the possible tumorgenic characteristic about TRIM52, gene set enrichment analysis (GSEA) was performed. Gene signature with the enrichment score positively associated with TRIM52 expression was selected from the MsigDB.

### Tissue specimens

Forty ovarian serous adenocarcinomas patients with FIGO stages of II–III were recruited. They were treated in the Department of Obstetrics and Gynecology, Tenth People’s Hospital, Tongji University (Shanghai, China) between 2013 to 2015. Tumor tissues and adjacent noncancerous tissues were collected for quantitative real-time PCR (qPCR) assays addressing TRIM52 and NF-kB P65 mRNA expressions. Pearson’s correlation analysis of TRIM52 and NF-kB P65 was subsequently performed. The design study was approved by the ethics committee of the Tenth People’s Hospital, Tongji and informed consents were signed by all patients prior to participation in the study.

To further explore TRIM52 expression in ovarian cancer, a IHC TMA (Alina Biotechnology co., LTD, Xi'an, China) containing 216 EOC and eight normal ovarian tissue was processed and stained with TRIM52 antibody (Novus, NBP2-31651). A total of 11.5, 35.9, 47.9 and 4.7% patients had stages I–IV disease, respectively, with the median age being 49 years. The results of immunochemical assays were scored by two reviewers. The positive staining percentage of <5, 5–25, 25–50, 50–75, and >75% were correspondingly scored as 0, 1, 2, 3, and 4.

### Cell lines

Five human ovarian cancer cell lines, OVCAR3, A2780, CAOV3, SKOV3, and HO8910 were involved in the study. Among them, OVCAR3 and CAOV3 were obtained from ATCC, and A2780; SKOV3 and HO8910 were from Chinese Type Culture Collection, Chinese Academy of Sciences. Cells were maintained in RPMI 1640 medium (A2780, SKOV3) or DMEM (HO8910, CAOV3, OVCAR3), supplemented with 10% fetal bovine serum, 100 U/ml penicillin sodium, and 100 mg/ml streptomycin sulfate at 37 °C in a humidified air atmosphere containing 5% CO_2_. Cells were used for following assays when they were in the logarithmic growth phase.

### RNA interference and lentiviral infection

To clarify the effectors of TRIM52 gene on ovarian cancer growth, the experiments of knockdown TRIM52 and overexpressing TRIM52 were performed. Three shRNAs targeting TRIM52 knockdown (position 585–603, GTTGTTCCAAGACCAAGAT, TRIM52-Ri1; position 756–774, AGAAATACTGGAAGCATAC, TRIM52-Ri2; position 975–993, GGGCATGTGCTTTAAACAC, TRIM52-Ri3) and one nonspecific scramble shRNA sequence as NC were cloned into a lentiviral vector PLKO.1 (Addgen). Then, the constructs of pLKO.1-shTRIM52 were co-transfected with Pspax2 and pMD2.G lentiviral packaging plasmids into HEK293T to produce a lentivirus by using lipofectamine 2000 (Life Technologies), according to the manufacture’s instruction. In addition, full-length human TRIM52 (NM_032765.3) targeting TRIM52 overexpression was cloned into the lentiviral vector pLVX-Puro (Clontech). Lentiviral empty vector was used as control. Similarly, pLVX-Puro-TRIM52 constructs were co-transfected with lentiviral packaging plasmids into HEK293T to produce a lentivirus. Viral supernatant was collected at 48 h after transfection and used to infect the cells. Stable pools were generated by puromycin (Sigma) selection for following assays. All constructs were identified through sequencing.

### RNA extraction and qPCR

mRNA levels of TRIM52, NF-kB P65, and NF-kB downstream effectors were detected by qPCR. Total RNA was extracted using TRIzol Reagent (Invitrogen) according to the manufacturer’s protocol. Total purified RNA was reverse transcribed with random primers using cDNA synthesis kit (Thermo Fisher Scientific, Rockford, IL, USA). By the resulting cDNA used as a template for qRT-PCR, real-time PCR using SYBR Green (Thermo Fisher Scientific, Rockford, IL, USA) was performed to detect the mRNA levels of the indicated genes. GAPDH was served as an internal control. The primer information is provided in Supplementary Table 1. All reactions were conducted on an ABI 7300 real-time PCR machine (Applied Biosystems, Foster City, CA, USA) using the following cycling parameters: 95 °C for 10 min, followed by 40 cycles of 95 °C for 15 s and 60 °C for 45 s. Quantitation of relative gene expression was calculated using comparative ^▵▵^Ct method. All data represent the average of three replicates.

### Western blot

Protein expressions of TRIM52, NF-kB P65, and NF-kB downstream effectors were tested by WB. Cell protein was extracted from stimulated ovarian cancer cells using RIPA buffer (50 mM Tris-HCl [pH 7.5], 150 mM NaCl, 1% Triton X-100, and 0.5% Na-deoxycholate) containing protease inhibitors. BCA protein assay kit (Thermo Fisher Scientific) was utilized to determine protein concentrations. Thirty micrograms samples of lysates were separated on 10–15% SDS-PAGE gels and transferred to PVDF membranes. The membranes were incubated with primary antibodies overnight at 4 °C. Primary antibody incubation was followed by incubation with an HRP-conjugated secondary antibody. Signals were detected using chemiluminescenct substrate (ECL, Bio-Rad, Richmond, CA, USA). Finally, the band intensity was measured using Image J software (NIH, Bethesda, MD, USA). Primary antibodies MMP9, Bcl2, caspase 3, IL8, TNFα, NF-kB P65, IKK, IKBα, and phosphorylated IKKβ and IKBα were purchased from Abcam, and antibodies against TRIM52 was from Novus. GAPDH and H3 serving as internal control were obtained from CST Biotech. (Danvers, MA, USA) and Abcam, respectively.

### Immunofluorescence

Immunofluorescence was employed to show TRIM52 expression in ovarian tumor tissue. Cells were pelleted, washed with PBS (phosphate-buffered saline), and fixed at room temperature using 4% paraformaldehyde for 30 min and permeabilized with 0.5% Triton X-100 for 10 min. Cells were sequentially stained in following order and condition: TRIM52 rabbit antibody (Novus) or NF-kB P65 rabbit antibody (Abcam) (1:100 overnight at 4 °C) and fluorescence secondary antibody (Bitianyun Company, China) (1:300 1 h at room temperature). Cells were washed with PBS between each antibody staining. Finally, cells were stained with DAPI (Bitianyun Company, China) (1:500) and mounted on slides using antifade mountant (Bitianyun Company, China). The slides were kept in the refrigerator under −20 °C. Images were acquired using fluorescence microscope (Leica Company).

### Cell migration and invasion assays

Cell migration and invasion were assessed by Transwell experiments. For cell invasion assay, the upper chamber of the transwell (Corning, NY, USA) was coated with Matrigel (BD Biosciences) at 37 °C in a 5% CO_2_ incubator for 30 min. Indicated cells were trypsinized, resuspended in serum-free DMEM medium, and placed in the upper chamber (5 × 10^4^ cells/well). Then, DMEM medium supplemented with 10% FBS was added to the lower chamber. The plates were incubated for 48 h. Cells in the upper chamber were completely removed with a cotton swab. Cells migrating into the lower chamber were washed with PBS, fixed in 4% paraformaldehyde, and stained with 0.5% crystal violet. Finally, the cells were counted under a microscope in five random fields. For cell migration assay, the experiment were performed under same conditions, except Matrigel was removed when the upper chamber was precoated.

### Flow cytometry for cell apoptosis

Flow cytometry was employed to evaluate cell apoptosis. Cells were harvested directly or 48 h after transfection and washed with PBS. Then, the cells were double-stained with Annexin V-fluorescein isothiocyanate (FITC) and PI in sequence at 4 °C for 30 min and protected from light for 5 min. Flow cytometry data collection was performed on FACScan instrument (Becton Dickinson, Mountain View, CA, USA) to evaluate cells apoptosis. The control experiment of cell apoptosis was carried out by adding ZVAD (an apoptosis inhibitor) into infected SKOV3 or CAOV3 cells.

### Cell proliferation assay

Cell proliferation assay was detected by using the Cell Count Kit-8 (CCK-8, Dojindo Laboratories) according to the manufacturer’s instructions. Indicated cells were seeded into 96-well plates at a density of 5 × 10^3^ cells per well, then were incubated with shRNA–TRIM52 lentivirus or synthetic TRIM52 lentivirus for 0, 24, 48, and 72 h. No scramble shRNA sequence and empty lentiviral vector were used as controls, respectively. Sequentially, CCK-8 solution (10 μL in 100 μL DMEM or RPMI 1640 medium) was added to each well and incubated 37 °C for 1 h. OD was recorded at wavelength 450 nm by a microplate reader (Bio-Rad). Each assay was performed in triplicate.

### NF-KB activation assays

To further explore whether the NF-kB signal pathway was involved in TRIM52-mediated regulation in ovarian cancer, TNF-α (an activator of NF-kB signal), LPS (an activator of NF-kB P65), and PDTC (an inhibitor of NF-kB P65) were employed. TNF-α or LPS was co-incubated with infected SKOV3 or CAOV3 cells. PDTC was added into overexpression TRIM52 HO8910 cells. The experiments addressing cells invasion, migration, apoptosis, and proliferation were performed as described in previous studies. Protein expressions of nuclear protein P65, plasmosin P65, and NF-kB downstream effectors were detected to evaluate the activation of the NF-kB signal.

### In vivo tumorigenicity assay

Animal experiments were approved by the Animal Experimentation Ethics Committee of Tenth People’s Hospital affiliated to the Tongji University. Female athymic Balb/c nude mice that were 5-week-old and weighed 20–22 g were provided by the Shanghai Laboratory Animal Company. The mice stayed in a pathogen-free animal facility and randomly assigned to the experimental or control group (six mice per group). SKOV3 cells infected by TRIM52-Ri3 virus were injected into the mouse axilla at 4 × 10^6^ each. No scramble sequence transfection was used in the control group. Non-infected SKOV3 cells were also employed to evaluate the tumor formation. The volume of the tumor was measured using calipers for 3 days. Mice were killed 33 days post inoculation when tumor nodules were near to the size of 400 mm^3^. Part of tumor was fixed in 4% paraformaldehyde and paraffin embedded to perform the following hematoxylin and eosin (HE) staining, Tunel staining, cleaved caspase-3 staining, and Ki-76 IHC assay. Other tissues were frozen in liquid nitrogen immediately for next western blot experiment to detect the protein expressions of NF-Kb and TRIM52.

### Statistical analysis

Data were acquired from at least three independent experiments as noted. Student’s *t*-test (two-tailed) was used to compare the two groups. Statistics was performed in GraphPad Prism7 (San Diego, CA, USA). *P-*values < 0.05 were considered significantly different.

## Electronic supplementary material

The primer information of qPCR experiments

TRIM52 expressions in different ovarain cancer cell lines

TNF-α induces the activation of NF-kB signal
