# Supplementary material for: TRIM52 plays an oncogenic role in ovarian cancer associated with NF-kB pathway
Source: Cell Death Dis. 2018 Sep 5;9(9):908. doi: 10.1038/s41419-018-0881-6 (PMC6125490; doi:10.1038/s41419-018-0881-6)
Supplement: Supplementary file 1 — The primer information of qPCR experiments [file 41419_2018_881_MOESM1_ESM.docx]

Supplemental Table 1

| Gene | Primer F | Primer R |
| --- | --- | --- |
| TRIM52 | 5' ATGGCTGGTTATGCCACTACT 3' | 5' CTCGTCCTCCTTACTCCACAG 3' |
| NFKBp65 | 5' GAATGGCTCGTCTGTAGTG 3' | 5' TGGTATCTGTGCTCCTCTC 3' |
| IL8 | 5' CAAGAGCCAGGAAGAAAC 3' | 5' TGGTCCACTCTCAATCAC 3' |
| TNFα | 5' GGTATGAGCCCATCTATCTG 3' | 5' AGGGCAATGATCCCAAAG 3' |
| MMP9 | 5' AAGGGCGTCGTGGTTCCAACTC 3' | 5' AGCATTGCCGTCCTGGGTGTAG 3' |
| BCL2 | 5' AGACCGAAGTCCGCAGAACC 3' | 5' GAGACCACACTGCCCTGTTG 3' |
| Caspase3 | 5' AACTGGACTGTGGCATTGAG 3' | 5' ACAAAGCGACTGGATGAACC 3' |
| GAPDH | 5' CACCCACTCCTCCACCTTTG 3' | 5' CCACCACCCTGTTGCTGTAG 3' |
