# Supplementary figures and images for: TRIM52 plays an oncogenic role in ovarian cancer associated with NF-kB pathway
Source: Cell Death Dis. 2018 Sep 5;9(9):908. doi: 10.1038/s41419-018-0881-6 (PMC6125490; doi:10.1038/s41419-018-0881-6)

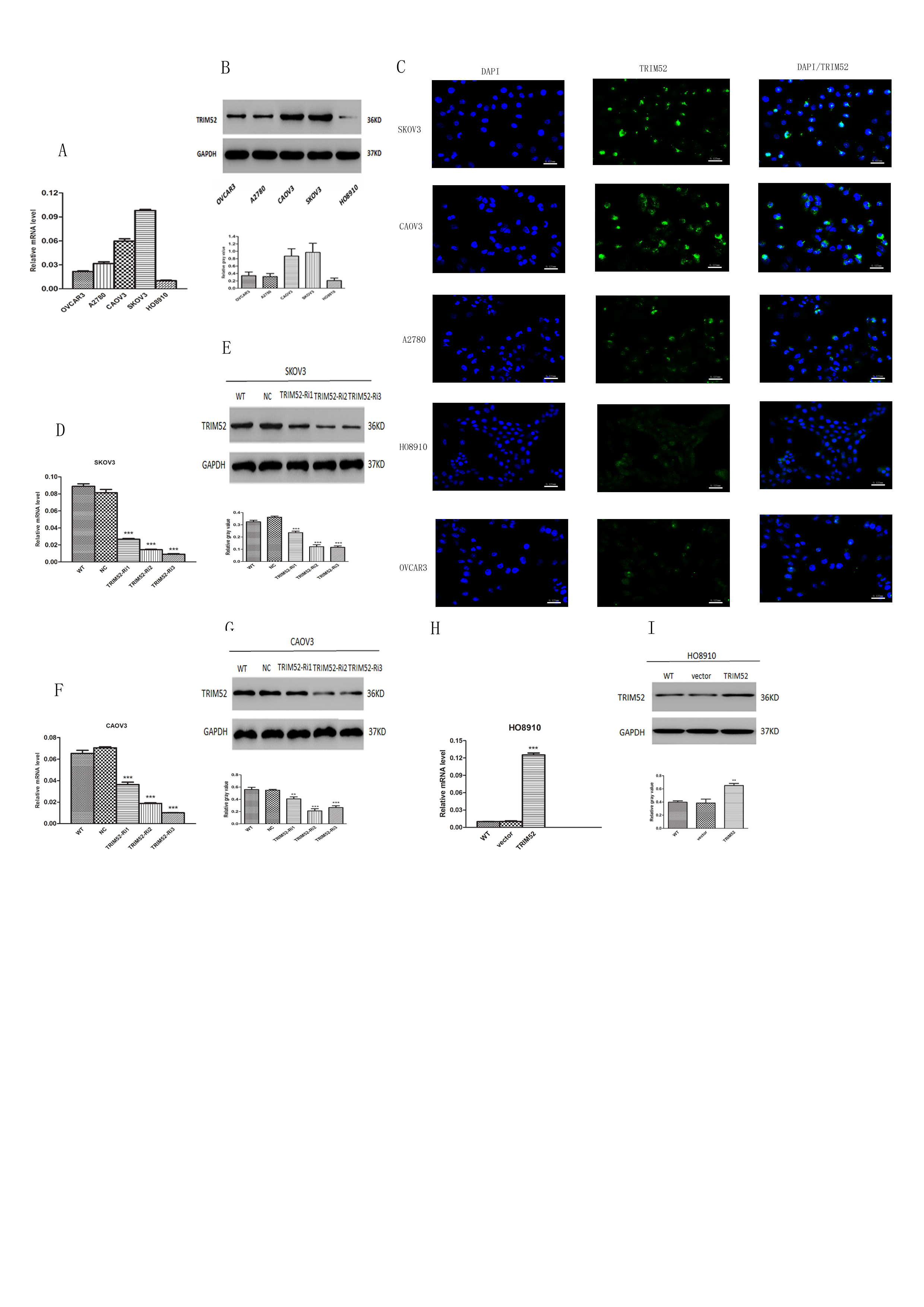

Supplement: Supplementary file 2 — TRIM52 expressions in different ovarain cancer cell lines [file 41419_2018_881_MOESM2_ESM.jpg]

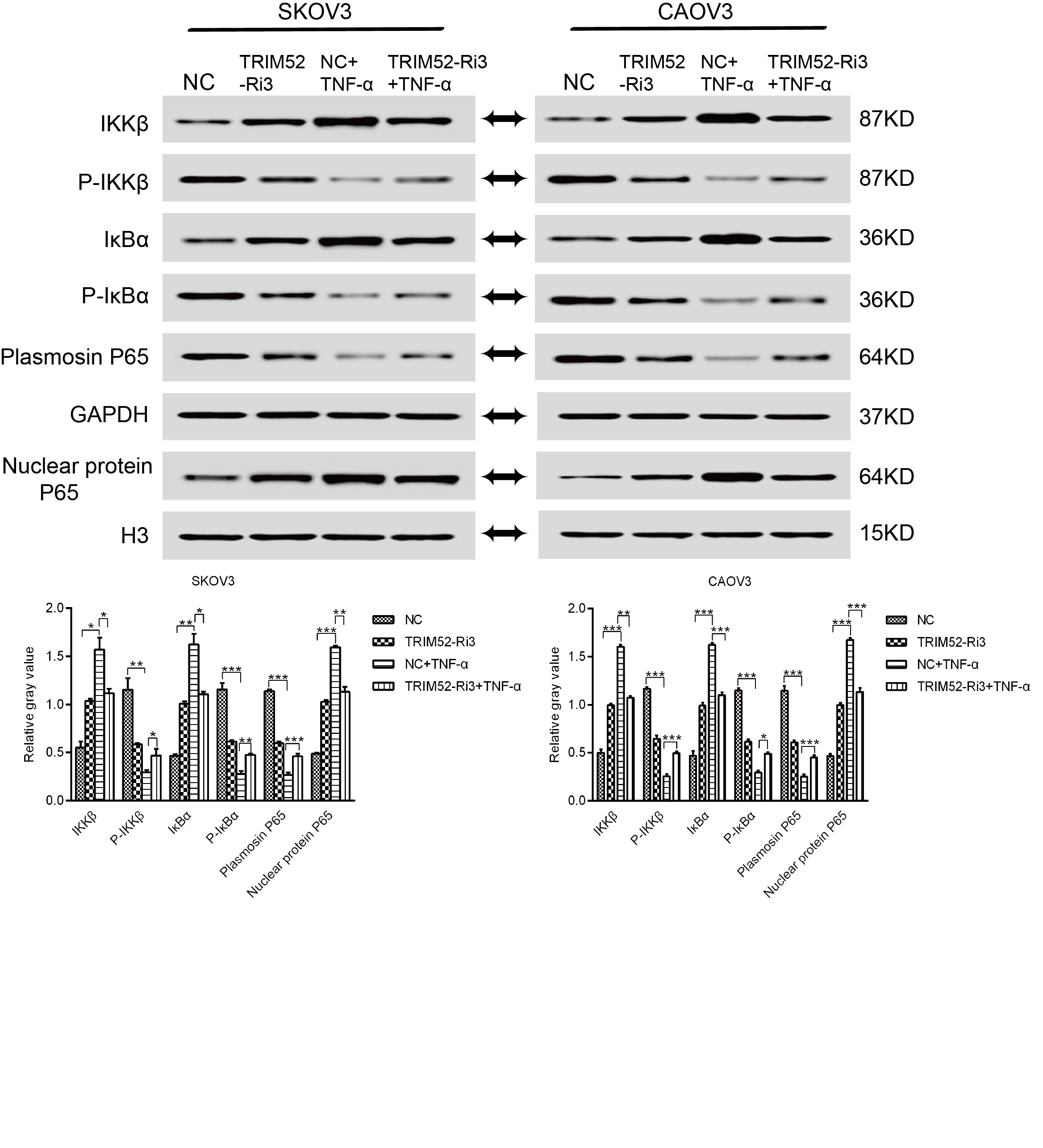

Supplement: Supplementary file 3 — TNF-α induces the activation of NF-kB signal [file 41419_2018_881_MOESM3_ESM.jpg]
